# Translational insights from EAE models : decoding MOGAD pathogenesis and therapeutic innovation

**DOI:** 10.3389/fimmu.2025.1530977

**Published:** 2025-05-20

**Authors:** Yanjia Zhang, Dong Li

**Affiliations:** ^1^ Tianjin Children’s Hospital (Tianjin University Children’s Hospital), Tianjin, China; ^2^ Department of Medicine, Tianjin University, Tianjin, China; ^3^ Department of Neurology, Tianjin Children’s Hospital (Tianjin University Children’s Hospital), Tianjin, China

**Keywords:** myelin oligodendrocyte glycoprotein antibody-associated disorder (MOGAD), experimental autoimmune encephalomyelitis (EAE), pathophysiology, innate immunity, adaptive immunity, treatment strategies

## Abstract

Myelin Oligodendrocyte Glycoprotein Antibody-Associated Disease (MOGAD) is a rare acquired demyelinating syndrome manifesting as optic neuritis (ON), transverse myelitis (TM), acute disseminated encephalomyelitis (ADEM), and brainstem encephalitis. The disease is characterized by serum autoantibodies targeting myelin oligodendrocyte glycoprotein (MOG), which is exclusively expressed on central nervous system (CNS) myelin and oligodendrocyte membranes. Experimental autoimmune encephalomyelitis (EAE) models have been instrumental in elucidating how these antibodies trigger complement-dependent cytotoxicity (CDC) and antibody-dependent cellular responses, leading to inflammatory demyelination. With most patients experiencing relapses and approximately 50% developing permanent disabilities, therapeutic strategies focus on reducing relapse frequency and severity. MOG-EAE models have directly informed acute treatment approaches including corticosteroids, plasma exchange (PLEX), and intravenous immunoglobulin (IVIG). Mechanistic studies in MOG-EAE models have revealed complex treatment responses and identified several translational targets, including complement inhibition, B-cell depletion strategies, and cytokine-directed therapies that are now advancing to clinical trials. Current immunosuppressive therapies include azathioprine (AZA), mycophenolate mofetil (MMF), and rituximab (RTX), with their differential efficacy in MOGAD versus MS and AQP4-NMOSD now explained by EAE model findings on distinct immunopathological mechanisms. Guided by EAE translational insights into MOGAD pathophysiology, ongoing clinical trials are evaluating novel targeted therapies including complement inhibitors, plasma cell-depleting agents, and antigen-specific tolerization approaches. These EAE-derived mechanistic insights are critical for developing personalized treatment strategies that address the unique immunopathology of this challenging condition.

## 1 Introduction

Myelin oligodendrocyte glycoprotein immunoglobulin G (MOG-IgG) antibody-associated disease (MOGAD) is a rare form of acquired demyelinating syndrome (ADS), distinguished by the presence of serum autoantibodies targeting myelin oligodendrocyte glycoprotein (MOG). This disorder is characterized by central nervous system (CNS) demyelination with mild axonal damage, concurrent with inflammatory cell infiltration, primarily composed of CD4+ T cells and granulocytes (1).MOGAD was not initially acknowledged as a distinct disease entity, but instead was viewed as a subtype or variant of multiple sclerosis (MS) or neuromyelitis optica spectrum disorder (NMOSD) (2, 3).This shift in perception exemplifies the progressively nuanced understanding of autoantibodies and their associated disorders within the realm of neuroimmunology.In 2011, Mader et al. published a seminal study that delineated the clinical characteristics of MOG antibody-positive patients and systematically differentiated them from MS and NMOSD patients (4). Subsequent research in this field progressed rapidly. In 2018, an international expert panel formally proposed MOGAD as a distinct disease entity, established its diagnostic criteria, and emphasized the crucial role of MOG antibody detection in diagnosis (5). The diagnostic differences, clinical aspects, and pathophysiological mechanisms of CNS demyelinating disorders are comprehensively summarized in **Table 1**.

**Table 1:** CNS demyelinating disorders comparative analysis.

Comparison Parameter	MS	MOGAD	NMOSD
**Diagnostic Criteria**	Based on McDonald criteria (dissemination of lesions in time and space) (6)131	• Presence of at least one typical clinical demyelinating CNS event (e.g., ON, TM, ADEM) • Positive serum MOG-IgG antibody test; borderline titers require supporting radiological or CSF features • Exclusion of alternative diagnoses including MS, AQP4-positive NMOSD, and infectious or neoplastic disorders (1)	AQP4-IgG positivity + core clinical features (e.g., optic neuritis, longitudinally extensive transverse myelitis) (7)
**Antibody Biomarkers**	No specific antibodies	MOG-IgG positive (approximately 97% of patients) (8)	AQP4-IgG positive (approximately 75% of patients) (9)
**Pathophysiological Mechanism**	T cells, B cells, and microglia orchestrate autoimmune processes that drive demyelination and neurodegeneration, resulting in axonal injury and sustained inflammatory response within the CNS (10, 11).	MOG antibodies bind to conformational epitopes of MOG protein, activating the complement system and inducing demyelination through CDC, ADCP, and ADCC mechanisms, characterized by perivenular primary demyelinating lesions, complement deposition, and infiltration of macrophages, microglia, CD4+ T lymphocytes, and neutrophils, ultimately resulting in neurological damage (12–17).	Anti-AQP4 antibodies attack astrocytes, initiating complement-mediated blood-brain barrier disruption and secondary demyelination (18, 19)
**Optic Neuritis Characteristics**	Unilateral, mild to moderate, relatively good recovery (20)	Uni- or bilateral, steroid-responsive, high recurrence risk (20)	Frequently bilateral, severe, poor visual recovery, frequent relapses (9)
**Spinal Cord Lesion Features**	Short-segment lesions (<3 vertebral segments) (21)	Both short and longitudinally extensive lesions possible (21)	Longitudinally extensive lesions (≥3 vertebral segments) (21)
**Brain Lesions**	Characteristic periventricular, callosal lesions	Subcortical white matter, brainstem, and cerebellar peduncle lesions (22)	Area postrema of medulla, hypothalamic lesions predominant, white matter lesions less common (23)
**Biological Markers**	CSF oligoclonal bands (>90% positive) (24)	Serum C3/C4 may be normal or slightly elevated in the acute phase, but these findings are not established diagnostic markers for MOGAD (25)	Decreased serum C3/C4 levels and elevated NLR (>2.86) may suggest NMOSD rather than MOGAD, though this is not yet an established diagnostic criterion (25, 26)
**Complement System Involvement**	No direct evidence of complement activation	Mild complement activation (C3, MAC deposition) (23)	Significant complement activation (C5b-9 deposition), efficacy of C5 (27)
**Treatment Strategy**	Disease-modifying therapies (e.g., β-interferons, fingolimod)	High-dose steroids in acute phase, some require immunosuppressive maintenance	Acute phase: steroids + plasma exchange; prevention: immunosuppressants (e.g., rituximab) or complement inhibitors (eculizumab)
**Comorbidities and Complications**	High comorbidity rate (depression, metabolic syndrome) (28)	Low T3 syndrome correlated with disease severity (22)	Frequently associated with other autoimmune diseases (e.g., Sjögren’s syndrome, systemic lupus erythematosus) (28)
**Prognostic Features**	Chronic progressive neurological deterioration	Relapsing-remitting, some patients with monophasic course, overall milder disability	High relapse rate, significant disability accumulation, worse prognosis with AQP4 positivity

This comprehensive table provides a systematic comparison of three major central nervous system demyelinating disorders: Multiple Sclerosis (MS), MOG Antibody-Associated Disease (MOGAD), and Neuromyelitis Optica Spectrum Disorder (NMOSD). The comparison encompasses diagnostic criteria, biomarkers, pathophysiology, clinical manifestations, imaging characteristics, treatment approaches, and prognostic indicators.

Bold text in the tables indicates the primary category or a general description for the corresponding row's data.

MOG protein, the target antigen of MOGAD, is predominantly expressed on the surface of oligodendrocytes in the outermost layer of central nervous system myelin (29). The molecular basis of its pathogenicity primarily stems from the β-sheet conformation of the MOG protein’s extracelluldomain, which forms a unique immunoglobulin-like structure, exposing multiple potential antigen-binding sites (30). The binding of MOG-IgG autoantibodies (predominantly of the IgG1 subtype (31)) produced by B cells to these exposed epitopes triggers a cascade of immune responses. These responses encompass: [1] complement activation leading to the formation of membrane attack complexes (4), [2] antibody-dependent cell-mediated cytotoxicity (32), and [3] T cell-mediated inflammatory responses (33). Furthermore, activated microglia and infiltrating macrophages secrete pro-inflammatory factors, exacerbating local inflammation (34). These complex pathological mechanisms collectively result in demyelination, oligodendrocyte death, and potential axonal damage (12). Crucially, the persistent inflammatory milieu and the presence of autoantibodies may impede the remyelination process, thereby impacting disease recovery (35). But the underlying pathogenic mechanisms, particularly the interplay between humoral and cellular immunity, remain incompletely understood.

MOGAD exhibits distinctive clinical, radiological, and pathological features compared to other types of ASD (36–39). Firstly, MOGAD manifests with a highly heterogeneous clinical presentation, predominantly encompassing phenotypes such as optic neuritis (ON), transverse myelitis, acute disseminated encephalomyelitis (ADEM), and brainstem encephalitis. These phenotypes can occur in isolation or in combination (40). Pediatric patients predominantly present with ADEM and optic neuritis, necessitating early recognition and prompt initiation of steroid therapy. Conversely, adult patients more frequently manifest with relapsing optic neuritis and myelitis, warranting particular vigilance regarding steroid dependence and relapse risk (41, 42). Secondly, MOGAD typically follows a relapsing-remitting disease course, which is potentially associated with fluctuations in antibody titers and dynamic changes in immune regulation (43). Notably, MOGAD patients often demonstrate a greater capacity for repair and more favorable prognosis compared to those with MS and NMOSD, potentially attributable to the distinct pathophysiological mechanisms underlying MOGAD (36).

In the absence of established MOGAD treatment guidelines, a comprehensive understanding of these mechanisms is imperative for guiding acute attack management, personalized symptom control, and long-term relapse prevention strategies. The current standard of care for acute MOGAD exacerbations involves intravenous high-dose methylprednisolone (IVMP), typically administered at 1g/day for 5 consecutive days (44). However, the risk of disease relapse increases significantly during the steroid tapering and discontinuation phase (45). For patients experiencing further clinical deterioration, plasma exchange or intravenous immunoglobulin (IVIG) may be considered as second-line therapeutic interventions (46). MOGAD therapeutic responses exhibit significant age-dependent variations. Regarding glucocorticoid sensitivity, pediatric patients respond favorably to high-dose corticosteroids during acute phases, although rapid tapering frequently precipitates relapses (41). Research indicates that children with ADEM phenotypes demonstrate steroid sensitivity, yet approximately 20% experience relapse following dose reduction (47). In contrast, while adult patients similarly exhibit marked responses to steroids during acute episodes, they face higher relapse risks, which may be independent of steroid tapering velocity (48). Some adult patients require adjunctive plasma exchange (PLEX) or intravenous immunoglobulin (IVIG) to manage severe exacerbations (49). Age-related distinctions also manifest in maintenance therapy selection, with pediatric patients preferentially receiving IVIG as long-term immunomodulatory treatment due to its superior efficacy compared to other immunosuppressants (such as azathioprine and mycophenolate mofetil) and its more manageable side effect profile (48). Adult patients, however, typically opt for rituximab (B-cell depleting agent) or alternative immunosuppressants, though their relapse prevention efficacy is less pronounced than in NMOSD, necessitating recognition that rituximab offers limited relapse control in certain adult patients (50).

The optimal maintenance therapy for MOGAD patients remains a subject of debate in the medical community. Frequently employed pharmacological interventions include oral corticosteroids, azathioprine (AZA), mycophenolate mofetil (MMF), and B cell-targeted biologics such as rituximab (RTX) and tocilizumab (TCZ). Studies have demonstrated that these agents can significantly mitigate the risk of disease recurrence and improve the annualized relapse rate (ARR) (51, 52).

Regarding relapse risk and treatment duration, approximately 20-34% of pediatric patients progress to relapsing disease courses, particularly when MOG antibodies persist. Oral corticosteroids administered for at least three months (≥0.16 mg/kg/day) reduce relapse risk by 88% (53). Adult patients exhibit substantially higher relapse rates (40-80%), especially within six months following initial presentation, thus requiring more aggressive maintenance therapy. However, standardized protocols remain elusive, and some patients may necessitate extended immunosuppression for several years (42).

Complementing pharmacological approaches, non-pharmacological interventions such as long-term functional rehabilitation, regular MOG antibody serological monitoring, and longitudinal MRI surveillance are equally crucial for pediatric MOGAD patients (54).Furthermore, in pediatric patients, MOG antibody titers typically exceed those observed in adults, potentially indicating more robust immune responses and necessitating more cautious tapering strategies (41). From an age-stratified treatment perspective, pediatric patients often benefit from IVIG and gradual steroid tapering to minimize long-term adverse effects (such as growth suppression and metabolic dysregulation) (48, 50). Adult patients require careful balancing of relapse control against medication toxicity, as rituximab may increase infection susceptibility, thus demanding individualized selection (48).

Experimental autoimmune encephalomyelitis (EAE) has long served as a pivotal animal model in neuroimmunological research, providing fundamental insights into the pathogenesis of various demyelinating disorders (55). The evolution of MOG-specific EAE models, particularly those incorporating human MOG-specific T cells and antibodies, has created unprecedented opportunities for investigating MOGAD-specific immunopathological mechanisms (56). Elucidating the parallels between EAE and MOGAD is crucial for unraveling the underlying pathogenic mechanisms and subsequently developing more effective and targeted therapeutic strategies.

This comprehensive review aims to address critical knowledge gaps by synthesizing experimental and clinical evidence across multiple mechanistic and therapeutic domains. Our primary objectives are threefold: First, to provide a detailed analysis of MOGAD pathogenic mechanisms as elucidated through the lens of EAE models, emphasizing novel insights into disease initiation and progression. Second, to critically evaluate current therapeutic strategies in light of experimental evidence, identifying mechanisms of action and potential areas for optimization. Third, to explore emerging therapeutic approaches based on recent mechanistic discoveries, with particular emphasis on targeted interventions that may offer improved efficacy and safety profiles for diverse patient populations.

In the context of rapidly evolving MOGAD research, this narrative review seeks to provide an integrated perspective on disease mechanisms and therapeutic approaches, bridging preclinical insights with clinical applications. We systematically analyze current mainstream therapeutic strategies and comprehensively summarize ongoing clinical trials, thereby offering valuable insights for both evidence-based clinical practice and translational research directions.

In conclusion, MOGAD’s age-dependent characteristics significantly influence treatment strategies: pediatric patients require focus on achieving complete remission of predominantly monophasic disease courses and minimizing steroid tapering risks, while adult patients necessitate reinforcement of long-term management for more frequently relapsing disease. Given the ongoing advancement of MOGAD-related research, this article provides a critical overview of current treatment strategies, anticipating that additional high-quality clinical studies, particularly randomized controlled trials, will furnish stronger evidence-based guidance for disease management.

## 2 Pathophysiology of MOGAD

Research on MOG protein has predominantly focused on its role as an autoantigen in EAE and MS (57, 58). However, MOG protein is now recognized as the principal target antigen in MOGAD (5). MOG protein is a crucial encephalitogenic protein, with expression confined to the outermost layer of CNS myelin and the plasma membrane of oligodendrocytes (59). Its extracellular domain exhibits high CNS specificity and can elicit both cellular and humoral immune responses (60, 61). In humans, MOG antibodies (MOG-Ab) exert pathogenicity by recognizing conformational epitopes of the MOG protein and forming bivalent interactions with its extracellular domain (13). This process can activate the complement system, leading to demyelination through complement-dependent cytotoxicity (CDC), antibody-dependent cellular phagocytosis (ADCP), and antibody-dependent cellular cytotoxicity (ADCC) mechanisms (14). Notably, while all MOG-IgG subclasses can induce ADCP, the MOG-IgG1 and MOG-IgG3 subclass autoantibodies are particularly potent in inducing CDC (14). Moreover, MOG protein itself can directly activate the classical pathway of the complement system by binding to complement components C1q and C3d, functioning as adhesion molecules, signaling molecules, or activators of the complement cascade (62). This process further amplifies the demyelinating effect.

The current understanding of MOGAD pathogenesis is largely derived from EAE (63, 64), and **Table 2** provides a detailed overview of the pathogenic mechanisms and therapeutic insights gained from these models. Additionally, neuropathological and clinical studies have provided corroborating evidence. The CNS pathology in MOGAD patients exhibits complex histological features. Lesions are primarily characterized by confluent primary demyelination surrounding small and medium-sized veins, accompanied by relative axonal preservation in both white and cortical matter, and reactive gliosis. Furthermore, significant complement deposition, along with infiltration and activation of macrophages and microglia, has been observed (12).The inflammatory infiltrate predominantly comprises CD4+ T lymphocytes and neutrophils, reflecting the immune-mediated nature of MOGAD (15). While MOGAD patients exhibit demyelination, the damage to astrocytes and oligodendrocytes is comparatively mild (15). This characteristic suggests fundamental differences in the pathological mechanisms between MOGAD and NMOSD. Moreover, clinical observations have revealed significant peripheral immune activation in MOGAD patients, contrasted with relatively less chronic inflammation within the CNS (12). This feature stands in stark contrast to multiple sclerosis (MS). These findings provide a theoretical foundation for treating MOGAD with plasma exchange (PLEX) or specific immunosuppressants (75), while also paving the way for novel research into targeted therapeutic strategies against specific inflammatory mediators or immune cell subpopulations.

**Table 2:** Pathogenic mechanisms and therapeutic implications from experimental autoimmune encephalomyelitis models in MOG-associated autoimmune disease.

Research Focus	Principal Findings	Underlying Mechanisms	References
**EAE Model Pathogenesis**	MHC II-dependent antigen presentation by B cells is essential for EAE pathogenesis	Full-length MOG protein with conformational epitopes (P42) activates B cells; MHC II-TCR interactions drive Th1/Th17 cell activation	(56, 65, 66)
**Pathogenicity of Anti-MOG Antibodies**	Antibodies promote demyelination and inflammation via triple mechanisms	FcγR-dependent microglial activation; complement activation; enhanced antigen presentation facilitating T cell activation	(59, 67, 68)
**Complement System Role**	Elevated complement activation products (SC5b-9, Ba) in MOGAD patients	Lower complement activation efficiency compared to NMOSD; potentially functions as an indirect inflammatory amplifier rather than essential pathogenic component	(67)
**Bidirectional B Cell Regulation**	Anti-CD20 therapy demonstrates contradictory effects in different EAE models	Dual functionality: pro-inflammatory (APC function) versus anti-inflammatory (IL-10 secretion by regulatory B cells)	(65, 69, 70)
**Novel Therapeutic Strategies**	Three emerging approaches targeting distinct immune pathways	Anti-CD19 (plasma cell depletion); FcRn targeting (IgG reduction); PLGA nanoparticles (Treg induction)	(68, 71, 72)
**Clinical Relevance**	Spontaneous RR-EAE in SJL/J mice more closely resembles human disease	MOGAD lesion resolution rate (72-79%) significantly higher than in NMOSD/MS, consistent with model characteristics	(73, 74)
**Biomarkers**	SIRI index effectively differentiates MOGAD from AQP4-NMOSD	Conformation-specific anti-MOG antibody detection represents the gold standard for MOGAD diagnosis	(68, 73, 74)

Bold text in the tables indicates the primary category or a general description for the corresponding row's data.

This table synthesizes key research findings in MOG-associated autoimmune diseases with emphasis on EAE models, highlighting how different EAE paradigms (protein-induced vs peptide-induced, SJL/J spontaneous models) have revealed distinct disease mechanisms. The table demonstrates the essential role of B cells as antigen-presenting cells in EAE pathogenesis, the pathogenic mechanisms of anti-MOG antibodies, and the model-dependent dual functions of B cells (pro-inflammatory vs regulatory). EAE findings provide translational insights into MOGAD’s unique immunopathology compared to MS/NMOSD, supporting diagnostic biomarker development and targeted therapeutic strategies that address specific immune pathways identified through these experimental models.

The EAE model provides a fundamental basis for understanding the immunopathological mechanisms of MOGAD; however, the clinical heterogeneity of MOGAD far exceeds the singular pathological manifestations observed in animal models. EAE is an autoimmune disease animal model induced by immunization with MOG protein, widely used in studying the pathological mechanisms of multiple sclerosis (MS). In comparing EAE with MOGAD, certain similarities exist, such as MOG serving as the target antigen in the EAE model, which aligns with the autoantibody target in MOGAD patients, suggesting that both conditions may share partial immune pathogenic mechanisms (76). Nevertheless, significant differences exist in their pathological mechanisms: MOGAD patients exhibit marked activation of the complement system (e.g., elevated C3 and C4 levels), whereas T cell-mediated inflammation predominates in the EAE model (77). Additionally, MOGAD patients demonstrate a significantly lower positivity rate of oligoclonal bands (OCB) in cerebrospinal fluid compared to MS patients (16.7% versus 94.2%), which differs markedly from the presentation in MS and its EAE model (77). Additionally, MOGAD patients demonstrate a significantly lower positivity rate of oligoclonal bands (OCB) in cerebrospinal fluid compared to MS patients (16.7% versus 94.2%), which differs markedly from the presentation in MS and its EAE model (78, 79). These differences indicate that although the EAE model helps us understand certain pathological aspects of MOGAD, more comprehensive research models are needed to elucidate the disease’s specific immune characteristics and clinical diversity. Future research should further explore the molecular-level similarities and differences between MOGAD and EAE to deepen our understanding of this disease’s heterogeneity.

The 2023 MOGAD expert consensus guidelines emphasize the critical importance of MOG-IgG1 testing in patients presenting with compatible clinical phenotypes (1). MOG-IgG titers exhibit significant correlation with patients’ clinical manifestations and relapse risk. Clinical studies demonstrate that patients with elevated MOG-IgG titers tend to present with more severe clinical symptoms and may exhibit more extensive CNS involvement, including concurrent optic nerve and spinal cord engagement (79). Furthermore, persistently elevated MOG-IgG titers are associated with a less favorable long-term prognosis, characterized by an increased risk of disability and higher frequency of disease relapses (80).

**Figure 1:**
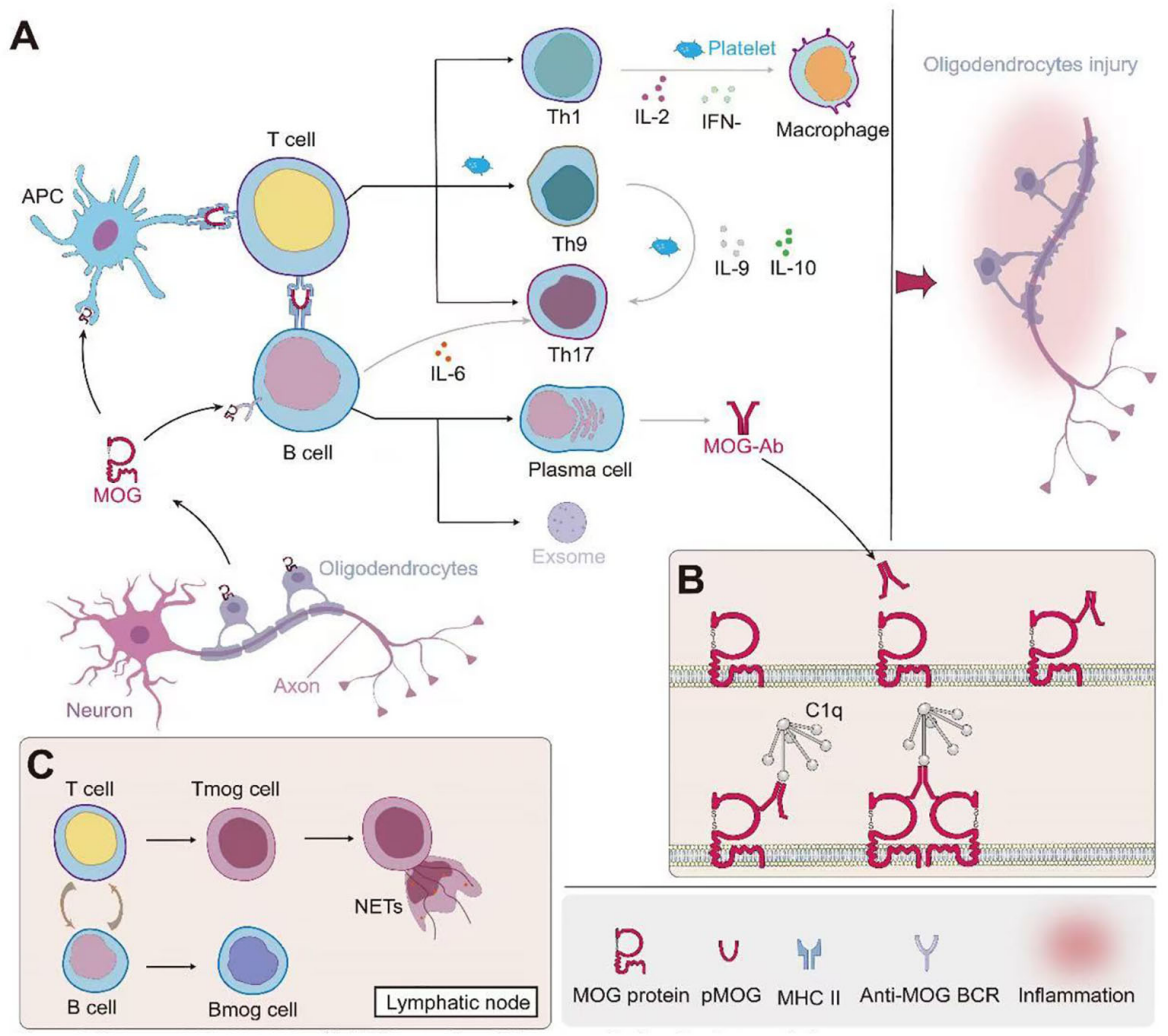
Immunopathogenesis of MOG-associated disease and oligodendrocyte injury. **(A)** Initiation and propagation of the immune response: MOG from oligodendrocytes is presented by antigen-presenting cells (APCs) to T cells. This leads to the differentiation of various T helper cell subsets (Th1, Th9, Th17) and the activation of B cells. Th1 cells produce IL-2 and IFN-γ, activating macrophages. Th9 and Th17 cells secrete IL-9 and IL-6 respectively, further modulating the immune response. B cells differentiate into plasma cells, producing MOG-specific antibodies (MOG-Ab). Platelets contribute to the inflammatory process. **(B)** Mechanism of MOG-Ab-mediated oligodendrocyte damage: MOG-Abs bind to MOG on the oligodendrocyte surface. This binding can lead to complement activation through C1q, resulting in the formation of the membrane attack complex (MAC) and subsequent cell lysis. Additionally, MOG-Ab binding may cause crosslinking of MOG proteins, potentially disrupting oligodendrocyte function. **(C)** Role of lymphoid organs: In lymph nodes, further interactions between T cells and B cells occur. This includes the formation of MOG-specific T cells (Tmog) and B cells (Bmog). Some activated T cells may form neutrophil extracellular traps (NETs), contributing to tissue damage. This cascade of events ultimately leads to oligodendrocyte injury, as depicted on the right side of panel A, potentially resulting in demyelination and axonal damage. The interplay between innate and adaptive immune responses, involving both cellular and humoral immunity, underscores the complexity of MOG-associated autoimmune processes.

Studies have demonstrated that MOGAD patients exhibit a higher probability of intrathecal MOG-IgG presence compared to other ADS (81). This phenomenon may be attributed to several factors: Firstly, MOGAD patients may exhibit more pronounced blood-brain barrier (BBB) disruption, facilitating antibody penetration into the CNS (17); Secondly, defects in the blood-spinal cord barrier at the central nervous system-peripheral nervous system (CNS-PNS) transition zones may contribute (82); Lastly, intrathecal synthesis (ITS) of MOG-IgG may also be a contributing factor (83). Clinical observations reveal that the degree of disability, cerebrospinal fluid (CSF) leukocyte count, and protein levels in MOGAD patients correlate with CSF MOG-IgG titers, but not significantly with serum MOG-IgG titers (81) Furthermore, studies indicate that patients with MOG-IgG ITS tend to exhibit more severe clinical courses, characterized by more pronounced pyramidal tract involvement and spinal cord lesions, with longitudinally extensive transverse myelitis being particularly prominent (83). The complex immunopathogenesis of MOGAD, leading to oligodendrocyte injury, is schematically depicted in **Figure 1**, setting the stage for a detailed examination of the contributions from B cells and plasma cells (Section 2.1), T cells (Section 2.2), and innate immunity (Section 2.3).

### 2.1 The role of B cells and plasma cells, in MOGAD

B cells and their terminally differentiated form, plasma cells, play a pivotal role in the pathogenesis of MOGAD through the production of MOG-Ab. Analogous to NMOSD, MOG-Ab are predominantly produced by plasmablasts and plasma cells in peripheral tissues (84). Studies have demonstrated that the inflammatory infiltrating B cell population primarily comprises B cells expressing CD79a and CD20, with a subset also expressing CD19 and the activation marker CD27 (85). Within the lesions, approximately 10% of lymphocytes are identified as CD38-expressing plasmablasts and CD138-expressing plasma cells. Of these, more than 90% contain IgG antibodies, potentially mediating disease progression (85). Besides MOG protein, B cell activation and function may be modulated by additional antigens, including butyrophilin (86) and erythrocyte membrane-associated proteins (87, 88).

The role of B cells in MOGAD pathogenesis extends beyond MOG-Ab production, encompassing several other crucial aspects. In MOG protein-induced EAE, B cells can function as APCs (66). The binding of B cell receptors (BCRs) to specific conformational epitopes of MOG protein, primarily involving proline 42, histidine 103, and serine 104 in the CC′ loop (31), triggers a cascade of biological effects: Firstly, it induces natural killer cell-mediated cytotoxicity; Secondly, it activates mitogen-activated protein kinase (MAPK) and protein kinase B (AKT) signaling pathways; Thirdly, it elevates intracellular calcium levels, leading to the activation of stress-related pathways; Lastly, these changes may compromise cellular cytoskeletal integrity (89). Additionally, B cells promote the proliferation of T helper 17 (Th17) cells through interleukin-6 (IL-6) production, thereby exacerbating MOG protein-induced EAE.

MOG-specific B cells (BMOG) exhibit dual functionality: Firstly, they are capable of presenting MOG antigens to MOG-specific T cells (TMOG); Secondly, they interact with TMOG in draining lymph nodes, effectively facilitating the production of autoantibodies (90). Studies demonstrate that BMOG exhibit significantly higher antigen presentation efficiency compared to conventional APCs, approximately 10,000-fold greater (91). However, no correlation has been observed between circulating BMOG and serum anti-MOG-Ab levels (92).

*In vitro* studies demonstrate that specific MOG-Ab significantly reduce transendothelial electrical resistance (TEER) in blood-brain barrier (BBB) models, directly confirming the capacity of MOG-Ab to compromise BBB integrity (93). Clinical studies have revealed an imbalance in immune cell subsets within the peripheral blood of MOGAD patients: On one hand, there is a reduction in the number of regulatory B cells producing interleukin-10 (IL-10); On the other hand, there is an elevation in levels of pro-inflammatory memory B cells and follicular helper T cells (Tfh), which promote the differentiation of B cells into memory cells and long-lived plasma cells (94).This imbalance in immune cell subsets may be a crucial factor contributing to the persistence of autoimmune responses in MOGAD.

Recent clinical studies have demonstrated significantly elevated levels of multiple immune-related factors in both the CSF and serum of MOGAD patients. These factors primarily fall into two categories: Firstly, activated complement proteins, including C3a, C5a, and Bb (95);Secondly, B cell-associated factors, encompassing α-proliferation-inducing ligand (α-APRIL), B cell activating factor (BAFF), and C-X-C motif chemokine ligand 13 (CXCL13) (96, 97). The elevation of these factors may potentially exacerbate neurological damage in MOGAD patients. This finding not only deepens our understanding of MOGAD pathogenesis but also identifies potential novel targets for future therapeutic strategies.

In conclusion, research indicates that B cells contribute to central nervous system (CNS) damage through multiple mechanisms, including (1): Release of potentially toxic exosomes (2), Secretion of pro-inflammatory cytokines (3), Antigen presentation to T cells, and (4) Production of autoantibodies (98). A significant imbalance in immune cell subsets has been observed in the peripheral blood of MOGAD patients, characterized by a decrease in regulatory B cells and an increase in memory B cells and circulating follicular helper T cells (Tfh). This dysregulation in immune cell proportions may be a critical factor exacerbating the autoimmune response (94). These findings not only enhance our understanding of MOGAD pathogenesis but also provide crucial theoretical foundations and potential targets for developing targeted therapeutic strategies.

### 2.2 The role of T cells, in MOGAD

Despite the low detection rate of MOG-specific T cells (TMOG) in clinical samples, studies suggest they may play a pivotal role in the autoimmune process of MOGAD (99). Research utilizing EAE models has identified CD4+ T cells as the predominant T cell subpopulation in MOGAD lesions (12). During the initiation phase of the disease, these CD4+ T cells release neutrophil extracellular traps (NETs), providing autocrine co-stimulatory signals for T cells (100). APCs process and present MOG protein (101), exposing epitopes composed of nine amino acid residues from the N-terminus of MOG protein on major histocompatibility complex class II (MHC II) molecules. These epitopes are subsequently recognized by TMOG, leading to T cell activation (102). Notably, MHC II molecules are also expressed on the surface of peripheral APCs. This finding suggests that exogenous MOG peptides may bind to and be presented by MHC II molecules without further antigen processing. This mechanism has significant implications for understanding peripheral nervous system (PNS) involvement in MOGAD patients (103).

Multiple effector T cell subsets (Th1, Th17, and Th9) independently induce EAE through distinct yet partially overlapping mechanisms (104). For instance, Th1 cells, driven by IL-12, secrete IFN-γ, which activates macrophages and promotes EAE progression (105). Upon migration to the CNS, myelin-specific Th1 and Th17 cells can induce demyelination and drive chronic inflammation (106). Th9 cells, under the influence of TGF-β and IL-4, produce IL-9 and IL-10, thereby promoting Th17 differentiation (107). Concurrently, clinical studies have revealed an imbalance in T cell subsets in MOGAD patients, characterized by increased proportions of Th1, Th2, and Th17 cells, as well as upregulation of regulatory T cell (Treg)-related cytokines (97, 108). This imbalance may play a crucial role in the pathogenesis and progression of the disease.

MOG-Ab, upon binding to T cells and activating the complement system, can damage oligodendrocytes (109) and specifically target myelin structures, resulting in extensive structural damage (110). This process further exposes antigens, inducing additional T cell recruitment (90), thereby creating a detrimental positive feedback loop. Simultaneously, B cells activate TMOG through antigen presentation, enhancing its ability to penetrate the BBB and subsequently compromising BBB integrity (90). Furthermore, MOG-Ab triggers the activation and proliferation of peripheral MOG-specific T cells in an Fc receptor-dependent manner, leading to damage to the PNS (68).

In summary, during CNS autoimmunity, the adaptive immune system launches a “double hit” on the brain through independent T cell and B cell effector mechanisms, resulting in severe tissue damage (111). This complex immunopathological mechanism elucidates the pathogenesis of MOGAD, providing crucial insights for understanding disease progression and developing potential therapeutic strategies.

### 2.3 Innate immunity in MOGAD

The innate immune system plays a crucial role in the progression of MOGAD. Despite the current lack of systematic studies, insights into the underlying mechanisms can be gained through EAE models. The pathological process of MOGAD involves multiple interrelated aspects, including antibody-mediated effects, complement system activation, inflammatory cell infiltration, and complex cytokine network regulation. The core pathogenic mechanism of MOGAD initiates with the specific binding of MOG antibodies to MOG proteins. This interaction triggers a cascade of immune responses, primarily including CDC, ADCP, and ADCC, ultimately resulting in oligodendrocyte damage (14). In the CDC process, complement C9 binds to IgG1 or IgG3 antibody-antigen complexes, initiating the classical pathway and forming the membrane attack complex (MAC), which directly induces cellular damage (14, 112). Notably, although complement activation in MOGAD is relatively minor (113), oligodendrocytes are more susceptible to complement attack due to their lower expression of surface complement regulatory proteins (such as complement receptor 1 (CR1), membrane cofactor protein (MCP), and H factor-related protein (HRF)) (114). Research has shown that disease relapse correlates more strongly with CDC and ADCP activity than with absolute MOG-IgG levels, a finding of significant clinical importance (14) Moreover, MOGAD patients exhibit significantly increased protein levels of activated classical complement pathway (CP) and alternative pathway (AP), which escalate with age (95).These findings have significant practical implications for the clinical management and development of personalized treatment strategies for MOGAD patients.

Biopsy and immunohistochemical analysis of brain tissue from MOGAD patients have revealed perivascular infiltration of various inflammatory cells, including microglia, macrophages, and neutrophils, predominantly concentrated around small and medium-sized veins in the vicinity of demyelinating lesions (12, 15).Notably, microglial infiltration within the cortex often extends beyond the demyelinating lesions (12), potentially elucidating the mechanism of lesion expansion. Clinical observational studies have further confirmed that MOGAD patients exhibit higher neutrophil-to-lymphocyte ratios (NLR) and platelet-to-lymphocyte ratios (PLR) compared to MS patients (115), as well as elevated levels of neutrophil-associated cytokines (such as interleukin-8 [IL-8] and granulocyte colony-stimulating factor [G-CSF]) (97). Moreover, unlike MS and NMOSD, the acute phase of MOGAD is characterized by upregulation of cerebrospinal fluid (CSF) myelin basic protein (MBP) rather than glial fibrillary acidic protein (116).

Neutrophils may play a crucial role in the pathogenesis of MOGAD. Studies on EAE and MS have shown that neutrophils mediate BBB leakage through the secretion of matrix metalloproteinases (MMPs) (117), and their IL-1β secretion may perpetuate inflammatory responses, leading to inflammatory damage in MS (118). As one of the first immune cells recruited from the blood to inflammatory sites, neutrophils exhibit both pro-inflammatory and anti-inflammatory properties, contributing to the balance of immune responses during inflammation (119).

Platelets also play a significant role in CNS inflammatory diseases. Studies have shown that platelets promote the proliferation and differentiation of MOG-specific autoimmune CD4+ T cells into T helper 1 (Th1) and T helper 17 (Th17) cells. Platelets secrete various cytokines, chemokines, and adhesion molecules, becoming key players in CNS inflammatory diseases by influencing leukocyte differentiation, migration, and infiltration (120).

Regarding the cytokine network, MOGAD patients exhibit significantly elevated levels of Th17-related cytokines (such as interleukin-6 [IL-6], IL-8, G-CSF, and granulocyte-macrophage colony-stimulating factor [GM-CSF]) in their CSF, along with altered levels of interferon-γ (IFN-γ), interleukin-10 [IL-10], and interleukin-1 receptor antagonist [IL-1Ra] (97). This distinctive cytokine profile, differing from that of MS, may reflect the unique immunopathological mechanisms of MOGAD.

The EAE model provides valuable insights into the pathogenesis of MOGAD. For instance, neutralization of interleukin-9 (IL-9) reduces mast cell infiltration in the CNS and ameliorates EAE symptoms (121). Dectin-1, a C-type lectin receptor, limits CNS inflammation in EAE and promotes beneficial myeloid cell-astrocyte interactions through oncostatin M-Osm receptor (OsmR) signaling (122). Moreover, IFN-γ plays a complex role in the pathogenesis of EAE. Interactions between IFN-γ and host CNS cells can selectively promote or inhibit neuroinflammation and pathogenesis (123). Interestingly, atypical EAE relies on interleukin-17 (IL-17) signaling, whereas classical EAE depends on GM-CSF and C-X-C chemokine receptor 2 (CXCR2) (124). Atypical EAE is associated with preferential upregulation of C-X-C motif chemokine ligand 2 (CXCL2) in the brainstem and CXCR2-dependent neutrophil recruitment (125).

## 3 Treatment of MOGAD

Currently, MOGAD treatment approaches are primarily based on clinical experience and extrapolation from other neuroimmunological diseases (particularly MS and AQP4-NMOSD). While large-scale randomized controlled trials (RCTs) specific to this patient population are lacking, observational studies and retrospective analyses have provided relevant evidence for MOGAD treatment.

### 3.1 Treatments for acute disease phases

Although acute-phase treatment may have limited impact on the long-term progression of MOGAD, timely and effective interventions are crucial for improving prognosis and delaying relapses (126). The primary objectives of acute-phase treatment include suppressing inflammatory responses, limiting central nervous system damage, and ultimately improving long-term neurological function.

Current clinical practice primarily relies on three treatment modalities: corticosteroids, plasma exchange, and intravenous immunoglobulin. Corticosteroids are the first-line treatment for acute MOGAD exacerbations, with the standard regimen typically consisting of intravenous methylprednisolone (IVMP) at a dose of 1g/day for 3–5 days. Research by Ramanathan et al. demonstrated that approximately 80% of patients respond favorably to this regimen (79). However, clinical practice and subsequent studies have revealed limitations of monotherapy with corticosteroids: it may be insufficient for severe attacks (21), and the risk of short-term relapse persists, necessitating consideration of preventive long-term treatment (43, 127–129). Furthermore, timing of treatment is critical. A retrospective study encompassing both AQP4-IgG+ NMOSD and MOGAD suggested that early intervention may lead to better outcomes (130). Notably, timely administration of IVMP in patients with ON can achieve near-complete recovery (21). A clinical study involving 42 patients demonstrated that slow tapering of steroids (up to six months) effectively reduced the risk of relapse (131). These findings underscore the necessity of individualized treatment plans and the importance of combining acute-phase interventions with long-term preventive strategies.Studies in MOG-specific EAE models have revealed complex therapeutic effects of high-dose dexamethasone (DXM, 50 mg/kg), demonstrating improved clinical symptoms but paradoxically enhanced neuroinflammation with cognitive impairment (132).This complexity highlights the critical importance of delivery strategies in glucocorticoid therapy. A novel approach utilizing acetalated dextran microparticles co-encapsulating MOG peptide and DXM demonstrated remarkable efficacy. Subcutaneous administration of these microparticles (MOG 17.6 μg, DXM 8 μg) at three-day intervals reduced clinical scores from 3.4 to 1.6, significantly outperforming conventional delivery methods. This enhanced therapeutic effect was accompanied by substantial suppression of disease-associated cytokines, including IL-17 and GM-CSF (133) Mechanistic investigations revealed that early DXM intervention not only attenuates clinical manifestations but also inhibits myelin and axonal degeneration while suppressing neuroinflammatory processes. Notably, DXM treatment enhanced mesencephalic astrocyte-derived neurotrophic factor (MANF) expression in spinal cord white matter. The therapeutic potential of MANF was further validated through intravenous administration, which improved early-stage EAE symptoms, suggesting its promise as a novel therapeutic target (134).

Plasma exchange (PLEX) is considered a crucial adjunctive therapy for patients with poor response to corticosteroids or severe disease. The standard PLEX regimen typically involves 5–7 sessions, administered every 1–2 days (135), or immunoadsorption (21). As early as 1999, research demonstrated the efficacy of PLEX for patients with severe demyelinating attacks who did not benefit from intravenous corticosteroids (46). An international survey by Whittam et al. further supported this view, indicating that approximately 70% of experts would opt for PLEX after failed steroid treatment (136). In a study of 50 MOG-ab positive patients, PLEX resulted in (near) complete recovery for 40% of patients, benefiting even those who failed IVMP treatment (21). Another study involving 65 pediatric ADS patients revealed that 72% exhibited moderate to complete functional recovery after PLEX, particularly those with ON and TM (137). However, variability in PLEX response may be related to treatment duration, suggesting that in some cases, PLEX might be prematurely discontinued (138). Intravenous immunoglobulin (IVIG), with its immunomodulatory and anti-inflammatory properties, also plays a significant role in the acute treatment of MOGAD. IVIG is particularly suitable for pediatric patients or those with contraindications to PLEX, with a standard regimen of 1–5 days and a total dose of 1-2g/kg (not exceeding 1g/kg per day). Research by Hacohen et al. confirmed the efficacy and safety of IVIG in pediatric MOGAD patients (40). More importantly, multiple studies have shown that IVIG can significantly delay short-term relapses and markedly reduce the annualized relapse rate (ARR) before and after treatment (139–141). For patients with severe attacks, high disability at nadir, or unclear response to IVMP, early escalation to PLEX or IVIG should be considered (21, 89, 142–144). However, the optimal timing for these escalation therapies lacks support from randomized controlled trial (RCT) data. Therefore, clinical decision-making often requires timely adjustment of treatment plans based on individual patient circumstances and disease progression, aiming to maximize acute-phase prognosis and long-term quality of life for MOGAD patients.

### 3.2 Long-term relapse prevention treatment: overall principles and objectives

The clinical management of MOGAD poses significant challenges, primarily due to its high relapse rate and potential for disability. Studies indicate that approximately 40% of adults and 30% of children experience disease relapse, with recurrent demyelinating episodes leading to varying degrees of neurological damage (44). More concerning is that about half of the patients may develop permanent disabilities, affecting vision, mobility, or sphincter function (52). These data underscore the urgency of developing effective long-term immunosuppressive treatment strategies. Concurrently, an ongoing MOGAD cohort study in China (ClinicalTrials.gov ID NCT06443333) aims to identify expression quantitative trait loci (eQTLs) specific to Chinese MOGAD patients, elucidating pathogenic genes and key mechanisms involved in the onset and progression of neuroimmunological diseases.

Currently, immunosuppressive therapies for MOGAD are largely based on experience with AQP4-NMOSD (40), with commonly used drugs including azathioprine (AZA), mycophenolate mofetil (MMF), and rituximab (RTX). Additionally, intravenous immunoglobulin (IVIG) and tocilizumab (TCZ) have shown potential efficacy (145–147). These treatment regimens have distinct characteristics and require selection and adjustment based on individual patient circumstances. The pharmacological profiles and radiological responses of these immunomodulatory therapies in MOGAD are further detailed in **Table 3**.

**Table 3:** Pharmacological profiles and radiological responses of immunomodulatory therapies in MOGAD.

Characteristics		Azathioprine (AZA)	Mycophenolate Mofetil (MMF)	Rituximab (RTX)	Maintenance IVIG	Tocilizumab (TCZ)
**Mechanism of Action**		Inhibits lymphocyte differentiation, antiproliferative effect (148)	Inhibits guanosine nucleotide synthesis, selective lymphocyte proliferation inhibition (149)	Anti-CD20 monoclonal antibody, B cell depletion (150)	Neutralizes autoantibodies, modulates T cell function	IL-6 receptor antagonist, inhibits inflammation
**Recommended Dose**		2–3 mg/kg/day (138)	1000–2000 mg/day, divided doses	1000 mg every 6 months, or based on CD19+ B cell count (151)	Maintenance: 0.4–2 g/kg every 2–8 weeks	8 mg/kg IV every 4 weeks; 162 mg SC weekly
**Time to Effect**		3–6 months (140)	3–6 months (140)	Quick, individual variation	Quick	Quick
**Efficacy**		Average ARR reduction 1.58, stabilizes EDSS (40, 140, 152–154)	73% relapse-free, ARR reduction 1.32 (155)	Reduces relapse rate, data varies (156)	Reduces ARR, suitable for children and pregnant women] (128)	Preliminary reduction in ARR, neurological improvement (79, 145)
**Relapse Risk**		50% may relapse	27% may relapse	Low relapse risk, varies	Low relapse risk (40, 140)	Low relapse risk, limited data
**Main Side Effects**		Bone marrow suppression, infection risk (151)	Bone marrow suppression, infection risk, teratogenicity (138, 141, 143, 144)	Infusion reactions, infection risk, neutropenia (151, 154, 156–159)	Headache, fever, infusion reactions, thrombosis risk	Infection risk, neutropenia, liver abnormalities, hyperlipidemia
**Adverse Reaction Rate**		24-33%	24-33%	Varies by dose	Low	More data needed
**Special Considerations**		Test TPMT before treatment; combine with corticosteroids	Caution for young females; corticosteroids initially (160)	Monitor CD19+ B cells [3rd-5th month] (151)	Possible long-term use	MOGAD application in research
**Monitoring Recommendations**		Monitor blood cell count, liver function	Monitor blood cell count, liver function	Monitor CD19+ B cells, adjust dosing	Monitor serum IgG, adjust dose	Monitor liver function, lipid levels, neutrophil count
**Comparative Radiological Responses** ***(combined with high-dose corticosteroid pulse therapy)***	**Optic Nerve Lesions**	• Reduction of nerve swelling and enhancement within 3–6 months (131) • Decreased T2 hyperintensity extent • Optic nerve atrophy reduced from 16% to 8% (52) • Decelerated RNFL thinning on OCT (131)	• Shortened inflammatory phase within 3–6 months (161) • Resolution of nerve swelling and enhancement (162) • Optic nerve atrophy reduced from 16% to 8% (79)	• 80% of patients show resolution of nerve swelling/enhancement within 3 months • 28% exhibit asymptomatic residual optic nerve atrophy • Reduced RNFL thinning progression	• 80% of patients show resolution of nerve swelling/enhancement within 3 months (163) • 24% exhibit asymptomatic residual optic nerve atrophy (164)	**Extrapolated from NMOSD data:** • Potentially rapid lesion resolution within 3–6 months (165) • Reduction in contrast-enhancing lesions (166)
	**Spinal Cord Lesions**	• Complete resolution of LETM in 77% of patients within 3 months (21) • Disappearance of cord swelling and enhancement (131) • Spinal cord atrophy rate: 5% (vs. 15% in untreated cohort) (52)	• Complete resolution of LETM in 77% of patients within 3 months (161) • Disappearance of cord swelling and enhancement (162) • 73% efficacy in preventing spinal cord relapse (162)	• Complete resolution of LETM in 77% of patients within 3 months (167) • Complete absorption of extensive (up to 15 segments) lesions (168) • Spinal cord atrophy rate: 5% (vs. 15% in untreated cohort) (169) • 14% breakthrough myelitis (primarily with B-cell repopulation) (167)	• Complete resolution of LETM in 77% of patients within 3 months (168) • Complete absorption of extensive longitudinal lesions (170) • Spinal cord atrophy rate: 5% (vs. 15% in untreated cohort) (163) • 14% breakthrough myelitis (primarily with insufficient dosing) (171)	• Potential attenuation of LETM via IL-6-mediated inflammation inhibition (172) • Possible prevention of syrinx formation • Estimated 93% efficacy in preventing spinal cord relapse (172)
	**Brain Lesions**	• Complete resolution in 83% of pediatric patients (54) • Reduction in new cortical/brainstem lesions (21) • Active lesion rate reduction from 0.5 to 0.2 per year (21)	• Complete resolution in 83% of pediatric patients (162) • Reduction in new cortical/brainstem lesions (79) • Active lesion rate reduction from 0.5 to 0.2 per year (79)	• Complete resolution in 83% of patients within 6 months (169) • Reduction in new brainstem/cerebellar peduncle lesions (173) • 61% potential breakthrough lesions due to immune escape (173)	• Complete resolution in 83% of patients within 6 months (164) • Reduction in new brainstem/cerebellar peduncle lesions (163) • 61% potential breakthrough lesions due to immune escape (163)	• Potential acceleration of lesion resolution and enhancement cessation • Reduction in new cerebral lesions • Attenuation of blood-brain barrier disruption (166)

This table summarizes the pharmacological characteristics and comparative radiological responses of immunomodulatory therapies for MOGAD treatment. Radiological outcomes presented reflect responses when treatments are administered in combination with high-dose intravenous methylprednisolone pulse therapy. Efficacy data are derived from retrospective observational studies and limited prospective trials, with superscript numbers indicating reference citations. Tocilizumab data are partially extrapolated from NMOSD studies due to limited MOGAD-specific evidence. Radiological improvement timelines and atrophy rates may vary based on lesion severity, treatment initiation timing, and individual patient factors.

Bold text in the tables indicates the primary category or a general description for the corresponding row's data.

### 3.3 Long-term relapse prevention treatment: old era

#### 3.3.1 Azathioprine and mycophenolate mofetil

Azathioprine (AZA), a first-line steroid-sparing immunosuppressive therapy, exerts its antiproliferative effect by inhibiting lymphocyte differentiation (148). The recommended dosage is 2–3 mg/kg/day, with full efficacy typically achieved after 3–6 months (138, 140). Multiple retrospective studies have demonstrated that AZA significantly reduces the annualized relapse rate (ARR) by an average of 1.58 and stabilizes EDSS scores (40, 140, 152–154). However, relapses are still observed in approximately 50% of patients, highlighting the need for treatment optimization. Notably, AZA should be initially combined with oral corticosteroids, as most relapses occur in patients not concurrently taking oral prednisone (21, 140) Concurrently, a phase 3 randomized, placebo-controlled trial evaluating AZA for relapse prevention is ongoing (ClinicalTrials.gov ID NCT05349006).

Mycophenolate mofetil (MMF) selectively inhibits B and T lymphocyte proliferation by suppressing *de novo* guanosine nucleotide synthesis (149). The typical dosage is 1000–2000 mg/day, administered in two divided doses. A meta-analysis revealed that approximately 73% of patients remained relapse-free after MMF treatment, with no significant difference in relapse-free rates between adults and children. MMF treatment reduced the mean ARR by 1.32 (155). Similar to AZA, MMF requires 3–6 months to reach full efficacy and should be initially combined with oral corticosteroids (140).

Although both treatment regimens demonstrate efficacy, they are associated with varying degrees of relapse risk and side effects. The primary side effects of AZA and MMF include bone marrow suppression and increased infection risk, with adverse reactions occurring in approximately 24-33% of patients (58, 133, 136–139, 174). For AZA, testing for TPMT activity prior to treatment initiation is recommended to identify patients at high risk for potentially fatal bone marrow suppression (151). Furthermore, MMF is teratogenic, requiring special consideration when used in young female patients (160).

#### 3.3.2 Rituximab

Rituximab (RTX) is a B-cell depleting monoclonal antibody that targets the CD20 antigen (150). In adults, the typical regimen consists of 1000 mg administered intravenously every 6 months, or individualized dosing based on CD19+ B-cell counts (151). Pediatric dosing protocols differ from those for adults (175). Studies have demonstrated that when employed as a first-line treatment, RTX is associated with a significantly higher reduction in relapse rates compared to alternative therapies (63% vs. 26%) (156). However, the efficacy of RTX in MOGAD appears to be less pronounced than in AQP4-IgG positive NMOSD (167). Recent studies suggest that a treatment regimen based on CD27-positive B-cell repopulation may be more effective. Additionally, FCGR3A gene polymorphism analysis can be employed to assist in evaluating RTX efficacy (176). RTX is associated with a spectrum of adverse effects, including leukopenia, infusion-related reactions, and hypogammaglobulinemia. These side effects lead to treatment discontinuation in approximately 13.71% of patients (151, 154, 156–159).

#### 3.3.3 Maintenance IVIG

Intravenous immunoglobulin (IVIG) may serve as an effective maintenance treatment option for specific patient populations, particularly children and pregnant women (128). However, the widespread application of this therapy is constrained by its high costs and limited availability (144). The typical IVIG treatment protocol consists of an initial loading dose of 0.4 g/kg daily for 5 consecutive days, followed by a maintenance regimen of 0.4–2 g/kg administered every 2–8 weeks. Compared to other conventional immunotherapies, maintenance IVIG therapy has demonstrated a significant reduction in the annualized relapse rate (ARR) (40, 140). Recent studies have demonstrated the safety and efficacy of subcutaneous immunoglobulin (SCIg) in preventing MOGAD relapses (177). SCIg offers several advantages, including better tolerability, the possibility of self-administration, and the option for home-based treatment when infusion services are available, potentially making it a more convenient therapeutic alternative.

#### 3.3.4 Tocilizumab

**Figure 2:**
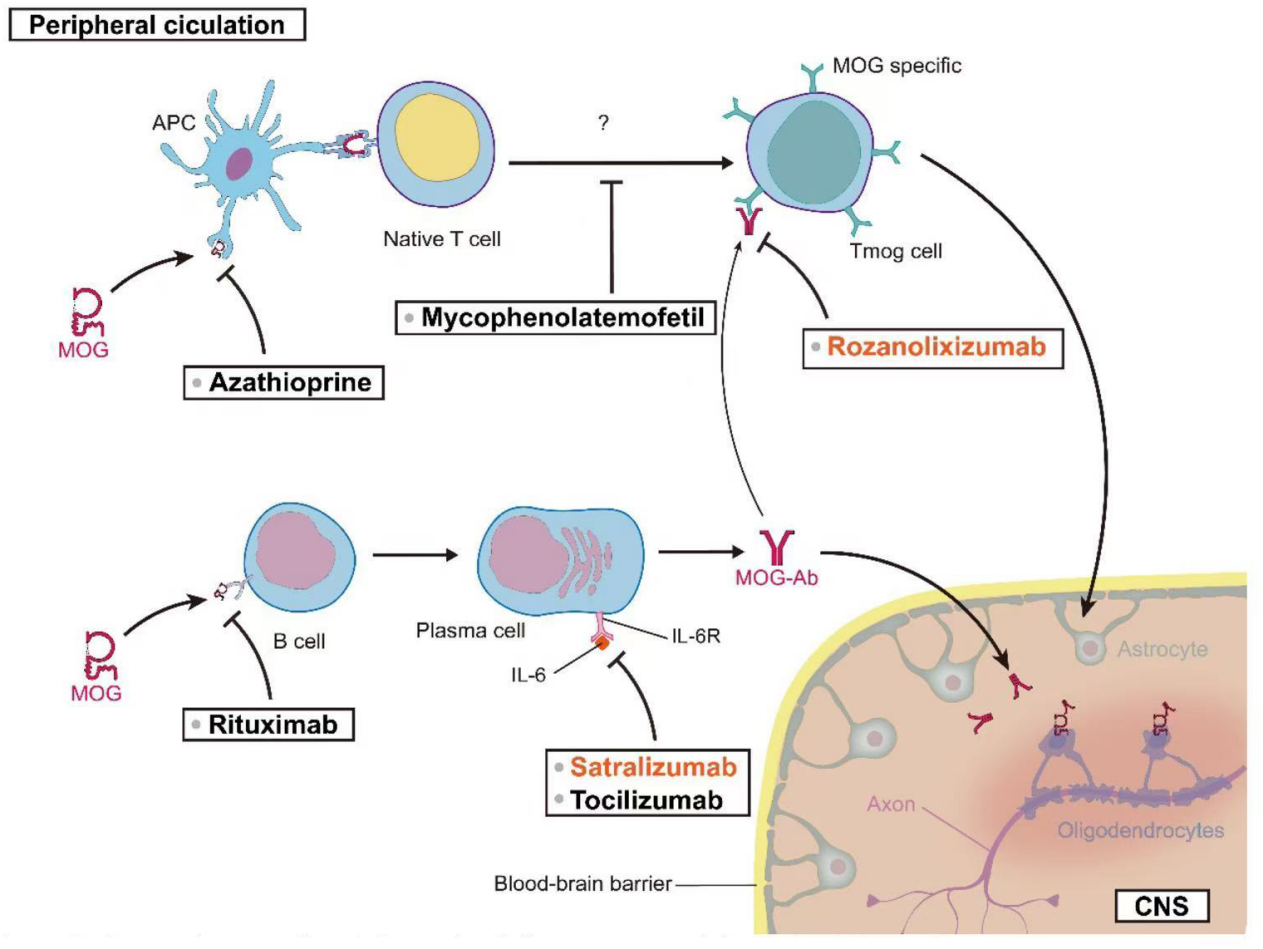
Therapeutic targets in MOG-associated disease: From peripheral circulation to CNS.

Tocilizumab (TCZ) has demonstrated notable efficacy in the treatment of refractory MOGAD patients. Small-scale case series studies suggest that TCZ may be highly effective for MOGAD patients who have been refractory to other immunosuppressive treatments (146, 178). The standard dosing regimen for TCZ in adults is 8 mg/kg administered monthly, with a maximum recommended dose of 800 mg/month. Two studies, collectively involving 19 pediatric patients, reported that 93% of patients (95% CI [54%–100%], I2 = 71%, p = 0.06) remained relapse-free during the follow-up period after initiating TCZ treatment. Prior to treatment, the ARRs in these two studies were 1.1 ± 0.4 and 1.78 ± 1.04, respectively. Post-treatment, the ARR in both studies decreased to 0. These results suggest that TCZ treatment significantly reduced the frequency of relapses in MOGAD patients (79, 145). A 2022 study by Ringelstein et al., involving 14 patients, provided data on TCZ’s impact on patient disability. The study utilized the Expanded Disability Status Scale (EDSS) to assess disability. Patients’ EDSS scores decreased from 2.75 ± 1.11 before TCZ treatment to 2.03 ± 1.26 after treatment. These results suggest that TCZ may contribute to improving patients’ functional status (146). To further validate the potential role of TCZ in MOGAD treatment, a randomized, controlled, multicenter study has been initiated (ClinicalTrials.gov Identifier: NCT06452537). This large-scale clinical trial is anticipated to provide more robust and comprehensive data regarding the efficacy of TCZ in MOGAD treatment. **Figure 2** offers a comprehensive visual guide to the therapeutic landscape in MOG-associated disease, delineating key intervention points along the immunopathogenic pathway from the peripheral circulation to the central nervous system. It illustrates how diverse strategies—targeting antigen presentation, T and B cell activity, pathogenic antibody clearance, and critical cytokine pathways like IL-6 signaling—aim to disrupt the disease cascade at multiple levels to ultimately reduce CNS inflammation and protect oligodendrocytes.

### 3.4 Long-term relapse prevention treatment: future era

#### 3.4.1 Rozanolixizumab

Rozanolixizumab (also known as Rozimab) is a high-affinity humanized immunoglobulin G4 (IgG4) monoclonal antibody that targets the human neonatal Fc receptor (FcRn). This antibody inhibits FcRn activity, resulting in rapid degradation of circulating antibodies, leading to a 70% reduction in antibody levels within 24 hours, an effect comparable to PLEX (179). Currently, rozanolixizumab is primarily indicated for the treatment of myasthenia gravis (180). In February 2022, the first phase 3 placebo-controlled randomized clinical trial for MOGAD was initiated (ClinicalTrials.gov Identifier: NCT05063162). This trial aims to evaluate the efficacy of rozanolixizumab in preventing relapses in MOGAD (181). Results are pending publication.

#### 3.4.2 Satralizumab

Satralizumab is a humanized immunoglobulin G2 (IgG2) monoclonal antibody produced in Chinese hamster ovary cells using recombinant DNA technology. It exerts its therapeutic effect by binding to both membrane-bound and soluble interleukin-6 (IL-6) receptors, thereby inhibiting the IL-6 signaling pathway (182). A phase III, randomized, double-blind, placebo-controlled, multicenter study is currently evaluating the efficacy, safety, pharmacokinetics, and pharmacodynamics of satralizumab (Enspryng^®^) as monotherapy or as an adjunct to baseline treatment in MOGAD patients (ClinicalTrials.gov Identifier: NCT05271409) (181). Satralizumab received its first global approval in Canada in June 2020 for the treatment of neuromyelitis optica spectrum disorder (NMOSD) in AQP4-IgG seropositive adults and children aged 12 years and older, demonstrating favorable outcomes (183).

#### 3.4.3 CT103A Cells

In recent years, cell-based therapies have garnered widespread attention in the field of autoimmune disease treatment. Chimeric Antigen Receptor T-cell (CAR-T) therapy, an innovative treatment approach, is being explored for various refractory diseases. In this context, a new clinical trial (ClinicalTrials.gov Identifier: NCT04561557) is evaluating the safety and efficacy of a novel CAR-T cell therapy utilizing CT103A cells for the treatment of relapsed/refractory antibody-mediated idiopathic inflammatory diseases. The CT103A CAR-T cell therapy employs genetic engineering techniques to modify T cells, enabling them to specifically recognize and eliminate B cells producing pathological antibodies. This approach aims to fundamentally reduce or eliminate the production of disease-causing autoantibodies, thereby achieving a therapeutic effect (184). While this clinical trial is not specifically targeting MOGAD patients, its outcomes may provide novel insights and approaches for MOGAD treatment.

#### 3.4.4 Calculus Bovis Sativus

Calculus Bovis Sativus (CBS), a traditional Chinese medicine, is documented in historical Chinese medical literature as possessing multiple therapeutic properties, including cardiac relief, phlegm resolution, choleretic effects, and sedative actions. Historically, CBS has been employed to treat a diverse array of conditions, including febrile coma, delirium, epilepsy, infantile convulsions, dental caries, pharyngitis, stomatitis, carbuncles, and furuncles (185). However, these traditional applications are predominantly based on empirical medicine and historical documentation, lacking systematic validation through modern scientific methodologies. To bridge the gap between traditional Chinese medicine theory and modern medical practice, researchers are investigating the potential applications of CBS in specific neurological disorders. Currently, an open-label clinical trial is underway to evaluate the efficacy and safety of CBS in the treatment of idiopathic inflammatory demyelinating diseases (ClinicalTrials.gov Identifier: NCT06474520). This study holds multifaceted significance: it exemplifies methodological innovation by integrating traditional Chinese medicine with modern clinical trial protocols, sets a precedent for the modernization of Chinese medicine research, and potentially offers novel therapeutic options for patients with idiopathic inflammatory demyelinating diseases. Paving the way for future advancements, **Table 4** provides a consolidated overview of the dynamic clinical trial landscape in MOGAD and related neuroimmunological disorders.

**Table 4:** Current clinical trials in MOGAD and related neuroimmunological disorders.

NCT Number	Study Title	Acronym	Study Status	Conditions	Interventions	Primary Outcome Measures	Enrollment	Phases
NCT06452537	Safety and Efficacy of Tocilizumab in Patients With MOGAD	TOMATO	Recruiting	MOGAD	DRUG: Tocilizumab DRUG: Prednisone	Time from randomization to the first MOGAD relapse as determined by an adjudication committee	102	PHASE2/3
NCT06474520	Efficacy and Safety of Calculus Bovis Sativus (CBS) for Idiopathic Inflammatory Demyelinating Disease	CBSinIIDD	Not Yet Recruiting	Idiopathic Inflammatory Demyelinating Disease, MS, NMOSD	DRUG: Calculus bovis sativus (CBS)	Modified Rankin Scale (mRS) within 12 weeks after treatment initiation	250	NA
NCT06541626	Sun Yat-Sen Cohort of CNS Idiopathic Inflammatory Demyelinating Diseases		Not Yet Recruiting	MS,NMOSD,MOGAD		Relapse, Long-term neurological function assessed using the EDSS	450	OBSERVATIONAL
NCT05271409	A Study to Evaluate the Efficacy, Safety, Pharmacokinetics, and Pharmacodynamics of Satralizumab in Patients With Myelin Oligodendrocyte Glycoprotein Antibody-Associated Disease	Meteoroid	Not Yet Recruiting	MOGAD	DRUG: Satralizumab, OTHER: Placebo	Time from randomization to the first occurrence of a MOGAD relapse in the DB treatment period	152	PHASE3
NCT05204459	MS-ResearchBiomarkerS	MS-ReBS	Recruiting	MS, NADs, Healthy Aging		Identifying risk factors for disability progression	1000	
NCT05063162	A Study to Evaluate the Efficacy and Safety of Rozanolixizumab in Adult Participants With MOGAD	cosMOG	Recruiting	MOGAD	DRUG: Rozanolixizumab, OTHER: Placebo	Time from randomization to first independently centrally adjudicated relapse	104	PHASE3
NCT04561557	Safety and Efficacy of CT103A Cells for Relapsed/Refractory Antibody-associated Inflammatory Diseases of the Nervous System	CARTinNS	Recruiting	Autoimmune Diseases of the Nervous System	BIOLOGICAL: CT103A cells, DRUG: Cyclophosphamide and fludarabine	Types and incidence of dose-limiting toxicity (DLT)	36	EARLYPHASE1
NCT06443333	National, Multicentric Registry Study on Neuroimmunological Diseases in China	NIDBase	Recruiting	MS, NMO, Myasthenia Gravis	Data collection and follow-up observation	Annual recurrence rate	7000	N/A
NCT06280755	Clinical Impact Through AI-assisted MS Care	RECLAIM	Not Yet Recruiting	MS, NMO	N/A	Data contribution	7000	N/A
NCT05605951	Acute Optic Neuritis Network	ACON	Recruiting	MS,NMO	Non-interventional study	Visual outcomes	200	N/A
NCT05545384	Immediate vs Delayed Treatment in Anti-MOG Syndromes	IDAR	Not Yet Recruiting	Acute Demyelinating Syndrome	Azathioprine, Rituximab	Annualized relapse rate	86	Phase 2/3
NCT05349006	Azathioprine in MOGAD	MOGwAI	Not Yet Recruiting	MOGAD	Azathioprine, Placebo	Time to first relapse	126	Phase 3

This table summarizes ongoing and planned clinical trials pertinent to Myelin Oligodendrocyte Glycoprotein Antibody-associated Disease (MOGAD) and related neuroimmunological disorders. It encompasses 12 trials, including interventional studies evaluating novel therapeutics such as tocilizumab, satralizumab, and rozanolixizumab, as well as observational studies and registries. The trials span various phases, from early Phase 1 to Phase 3, and include both adult and pediatric populations. Key information provided includes NCT numbers, study titles, acronyms, enrollment targets, and primary outcome measures. This comprehensive overview offers insights into the current landscape of MOGAD research, highlighting emerging treatment strategies and efforts to enhance understanding of disease progression and management.

NA, Not Available.

## 4 Conclusions and future perspectives

Translational investigations linking EAE models and MOGAD have significantly progressed our knowledge of disease mechanisms and potential therapeutic goals. In recent years, the pathophysiological mechanisms of MOGAD have been elucidated to a great extend upon lessons learned from EAE models. These experimental results have, in turn, led to lead therapeutic innovations including engineered glucocorticoids and the discovery of novel therapy candidates such as MANF. Systemic evaluation strategies, and borrowing therapeutic approaches from MS and NMOSD have opened up new possibilities for the treatment of MOGAD. The advent of different treatment options, such as monoclonal antibody therapies and cellular-based treatments offers hope for MOGAD patients but needs to be confirmed in future adequately powered clinical trials prior implementing their use widespread.

The integration of EAE models with clinical MOGAD research is of paramount importance for elucidating the pathological mechanisms underlying this disorder. Regarding pathogenesis, EAE models have definitively demonstrated that MOG-specific T cells are essential for disease initiation, while anti-MOG antibodies merely exacerbate demyelination. In contrast, clinical investigations of MOGAD suggest that anti-MOG antibodies may directly participate in the pathogenic process, exemplified by their capacity to disrupt the microtubule cytoskeleton in oligodendrocytes (50, 186). Therefore, when synthesizing findings from both research domains, it is crucial to differentiate between the predominant role of T cells in EAE and the potential direct pathogenic effects of antibodies in MOGAD, while concurrently exploring cooperative mechanisms such as T-B cell interactions in human disease (187, 188). Concerning the controversial role of antibody pathogenicity, although antibodies are not requisite in EAE models, studies have revealed that serum from MOGAD patients can aggravate demyelination in animal models. This observation indicates the necessity for further validation of antibody pathogenicity in human disease through clinical research, such as analyzing correlations between antibody titers and disease activity (50, 189). Notably, transgenic mouse models expressing human MOG have provided valuable platforms for investigating the pathogenicity of human anti-MOG antibodies, further supporting the observation that MOGAD patient serum can exacerbate demyelination in experimental settings (190).

In comparing animal models with clinical phenotypes, EAE models typically manifest as acute monophasic disease courses, whereas approximately 50% of MOGAD patients exhibit relapsing disease trajectories (144). Radiological investigations have revealed that T2 lesions in MOGAD demonstrate greater propensity for complete resolution compared to those in NMOSD and MS, resembling the reversibility of acute inflammation observed in EAE (191). This similarity suggests that EAE models may be valuable for investigating acute-phase mechanisms, though they must be complemented with longitudinal observations that account for the chronic relapsing characteristics of MOGAD. Beyond EAE, toxin-induced demyelination models (such as those utilizing Pseudomonas aeruginosa lipopolysaccharide and lysophosphatidylcholine) have contributed significant insights into myelin repair mechanisms in MOGAD. Particularly, these models demonstrate that remyelination capacity may be more robust in MOGAD compared to MS, which aligns with the clinical observation that MOGAD T2 lesions show greater tendency for complete resolution compared to those in NMOSD and MS (192).Furthermore, MOGAD patients frequently present with multifocal central nervous system involvement, while optic neuritis and myelitis predominate in EAE models (144). This discrepancy indicates the necessity of incorporating multifocal pathology in experimental model design to more accurately recapitulate human disease phenotypes.

Translational research on therapeutic strategies demonstrates that B-cell depletion is ineffective or potentially disease-exacerbating in EAE models, whereas clinical studies of MOGAD indicate that rituximab (anti-CD20) may be efficacious in a subset of patients (144, 193). This discrepancy underscores the necessity for comprehensive analysis of functional differences among B-cell subpopulations (such as plasma cells) in disease pathogenesis, and for exploration of novel B-cell-targeted therapies, including Bruton’s tyrosine kinase inhibitors (194). Regarding antibody-targeted therapeutics, EAE model investigations have demonstrated that anti-FcRn antibodies can reduce IgG circulation, ameliorate neurological dysfunction, and improve visual function, thereby providing a theoretical foundation for clinical MOGAD treatments (such as efgartigimod) (189, 195). However, these findings require further validation through rigorous clinical trials.

In the domain of biomarker development for diagnosis and prognosis, cerebral lesions in MOGAD predominantly involve cortical and subcortical regions, whereas periventricular lesions characterize MS (196, 197). EAE models can be utilized to simulate specific lesion patterns (such as optic neuritis) and, in conjunction with high-resolution MRI, validate human imaging biomarkers (such as “H-type” spinal cord lesions) (144). Concurrently, optimization of anti-MOG antibody detection methodologies (comparing live-cell versus fixed-cell assays) and stratification studies correlating antibody titers with clinical phenotypes (such as monophasic versus relapsing disease) hold significant clinical relevance (144, 189).

Regarding the investigation of cooperative mechanisms and disease model optimization, T-B cell cooperative pathogenesis has been documented in EAE models, and analogous immune interactions may exist in MOGAD patients (188). The establishment of humanized murine models (such as those incorporating transplanted patient T and B lymphocytes) to simulate the human immune microenvironment facilitates comprehensive understanding of these mechanisms (187). Additionally, given that EAE predominantly represents acute disease manifestations while the progression mechanisms of MOGAD remain incompletely elucidated, the development of chronic or relapsing-remitting EAE models, in combination with passive transfer of anti-MOG antibodies, may more accurately recapitulate human disease progression (187, 194).

In conclusion, the integration of EAE and MOGAD research necessitates careful consideration of both similarities and differences between experimental models and clinical manifestations, with particular emphasis on T-B cell interactions, validation of antibody pathogenicity, and translation of therapeutic strategies. Through interdisciplinary collaboration encompassing fundamental immunology, neuroradiology, and clinical trial design, advancements in mechanistic elucidation and precision therapeutics for MOGAD can be substantially accelerated.

There are many challenges on the horizon in MOGAD that need to be addressed, but we anticipate a rapidly changing therapeutic landscape. Given the clinical heterogeneity of MOGAD and limitations in current EAE models to capture all aspects of human disease, continued experimental optimization is required. It is essential that EAE models mimicking MOGAD-specific pathogenic mechanisms be developed, particularly in developing platforms that incorporate human MOG-specific immune responses. Fundamental scientific investigation should push the boundaries of our understanding into molecular processes underlying MOGAD, including production and implications of the MOG IgGs by expansion in B cells as well as functions both regulatory on T-cells and within CNS inflammatory cascades. This knowledge has to be translated into therapeutic targets or biomarkers, and clinical research should combine evaluation of new therapies as well as predictive strategies for differential responsiveness to treatment.

We need to work with researchers, clinicians and patient communities across disciplines. Through enhanced international cooperation and continuous innovation in research methodologies, we anticipate significant improvements in personalized treatment approaches for MOGAD patients. Our ultimate goal extends beyond symptom management to developing comprehensive strategies for preventing disease onset and halting progression. These concerted efforts aim to provide more effective personalized treatment regimens for MOGAD patients, potentially leading to better quality of life and long-term prognosis in the near future.
